# Heme *b* distributions through the Atlantic Ocean: evidence for “anemic” phytoplankton populations

**DOI:** 10.1038/s41598-020-61425-0

**Published:** 2020-03-12

**Authors:** Evangelia Louropoulou, Martha Gledhill, Eric P. Achterberg, Thomas J. Browning, David J. Honey, Ruth A. Schmitz, Alessandro Tagliabue

**Affiliations:** 1 GEOMAR Helmholtz Centre for Ocean Research Kiel, Kiel, Germany; 2 Institute for General Microbiology, Christian-Albrechts-Universität, Kiel, Germany; 3 School of Ocean and Earth Science, University of Southampton, Southampton, UK; 4 School of Environmental Sciences, University of Liverpool, Liverpool, UK

**Keywords:** Biogeochemistry, Ecology, Environmental sciences, Ocean sciences

## Abstract

Heme *b* is an iron-containing cofactor in hemoproteins that participates in the fundamental processes of photosynthesis and respiration in phytoplankton. Heme *b* concentrations typically decline in waters with low iron concentrations but due to lack of field data, the distribution of heme *b* in particulate material in the ocean is poorly constrained. Here we report particulate heme *b* distributions across the Atlantic Ocean (59.9°N to 34.6°S). Heme *b* concentrations in surface waters ranged from 0.10 to 33.7 pmol L^−1^ (median = 1.47 pmol L^−1^, n = 974) and were highest in regions with a high biomass. The ratio of heme *b* to particulate organic carbon (POC) exhibited a mean value of 0.44 μmol heme *b* mol^−1^ POC. We identified the ratio of 0.10 µmol heme *b* mol^−1^ POC as the cut-off between heme *b* replete and heme *b* deficient (anemic) phytoplankton. By this definition, we observed anemic phytoplankton populations in the Subtropical South Atlantic and Irminger Basin. Comparison of observed and modelled heme *b* suggested that heme *b* could account for between 0.17–9.1% of biogenic iron. Our large scale observations of heme *b* relative to organic matter provide further evidence of the impact of changes in iron supply on phytoplankton iron status across the Atlantic Ocean.

## Introduction

Iron constitutes one of the most important nutrients for phytoplankton^1^ because it is a component of biomolecules, termed cofactors, that participate in fundamental metabolic processes such as photosynthesis, respiration, nitrogen and sulphate assimilation^2,3^. The iron-containing cofactors are grouped based on the coordination and chemical bonds of iron with other elements within the molecule, and one major group of iron cofactors are hemes^4^.

Hemes are iron-containing porphyrins of different structures that act as cofactors (i.e. prosthetic groups) in hemoproteins^5^, and are produced via the tetrapyrrole synthesis pathway^5^. Ambient iron concentrations control heme biosynthesis and thus intracellular heme concentrations^4^. Heme *b* (iron protoporphyrin IX) is considered the most common heme structure within an organism^6^ and is a constituent of the *b* type cytochromes, catalases, peroxidases, cytochrome P450, globins and nitrate reductase^7,8^. Hence, heme *b* is involved in electron transport and catalysis of hydrogen and other peroxides as well as oxygen control, oxygen-storage and oxygen-transport^8^. Some marine bacteria are known to use dissolved hemes in seawater as a direct source of iron^9–11^. In cyanobacteria, rhodophytes and cryptophytes, hemes are further metabolised to phycobilins via heme oxygenase^12^. Phycobilins function as chromophores in the light-harvesting phycobiliproteins and the photoreceptor phytochrome^12,13^.

Hemoproteins make up approximately 40% of the intracellular iron pool in phytoplankton, with hemoproteins containing heme *b* contributing towards approximately half of this amount^14^. Honey, *et al*.^15^ demonstrated that heme *b* accounts for between 1 to 40% of the total biogenic iron pool in marine phytoplankton (mean 18 ± 14%). At low iron concentrations in culture media (≤0.50 nmol L^−1^), 6 to 26% of the available iron was utilized for production of heme *b* cofactors for species from temperate ocean areas^15^. This percentage ranged from 0.2 to 16% for species from high latitude ocean regions^16^. These findings suggested that, similarly to iron-use efficiencies^14^, the heme *b* quota required for growth is also variable amongst species and growth conditions^15,16^.

To date, several studies have documented that low oceanic iron concentrations (<0.20 nmol L^−1^) lead in general to a decrease in growth, photosynthesis and nitrogen fixation rates of phytoplankton^3,17–22^. Although a number of studies have documented the declines in the abundance of several iron proteins in cultured phytoplankton^23–30^, only a few studies report the abundances of such proteins in the field^31–35^.

Iron deficiency in higher organisms is expressed by decreases in hemoprotein levels (e.g. hemoglobin). This condition is commonly described as “anemia” which literally refers to heme-iron deficiency. In a similar manner, reduced iron supply in the surface waters in the ocean leads to anemic phytoplankton. Hence, in both cultured and field phytoplankton populations, the concentrations of heme *b* tend to fall below 1.0 pmol L^−1^ under low iron (≤0.5 nmol L^−1^) conditions^15,16,35,36^. However, certain eukaryotes (*Phaeocystis, Chaeotoceros, Rhizosolenia spp*.) appear able to maintain growth despite the low heme *b* concentrations^16,36^. These species are considered to regulate and reduce intracellular heme *b* concentrations by allocating the available iron away from the hemoprotein pool in order to maintain other metabolic processes^16,36^. Heme *b* regulation is considered a response of phytoplankton to declining ambient iron concentrations that depends on species- specific requirements in iron and thus may be observed in the field during the iron-induced shifts^37^ from larger- to smaller-sized phytoplankton populations^36^.

In this study we present an extensive dataset of heme *b* abundance in the Atlantic Ocean. We synthesise previously published^15,16,36^ and new field data covering areas from the subpolar North Atlantic to the subtropical South Atlantic. Since the factors driving the heme *b* distribution in the natural environment are still uncertain, our aim was to examine physical, chemical and biological processes that potentially influence heme *b*. The Atlantic Ocean is a good study region for this purpose as it consists of several provinces that exhibit contrasting abundances of iron and macronutrients. In particular, the Atlantic Ocean is an area of scientific interest as it is subject to large spatial variability in iron supply^38,39^ with potential impacts on primary productivity, nitrogen and phosphorus cycling^40–45^, and carbon export^46^. Furthermore, low iron supply in the Atlantic Ocean has been connected to reductions in primary productivity^47,48^, oceanic CO_2_ uptake rates by phytoplankton^48^ and nitrogen fixation rates^41^ with potential consequences for the global climate^49^.

Finally, taking into account the laboratory experiments that showed that heme *b* makes up a significant portion of the total biogenic iron pool in marine phytoplankton, heme *b* could potentially provide an assessment of iron utilization *in situ*. Hence, the second part of our study examines (1) the comparability of field heme *b* concentrations to predicted heme *b* from the total biogenic iron pool using a global biogeochemical model, and (2) the potential utility of heme *b* as an indicator of the magnitude of the biogenic iron pool in the field.

## Results and discussion

### Study region

**Figure 1:**
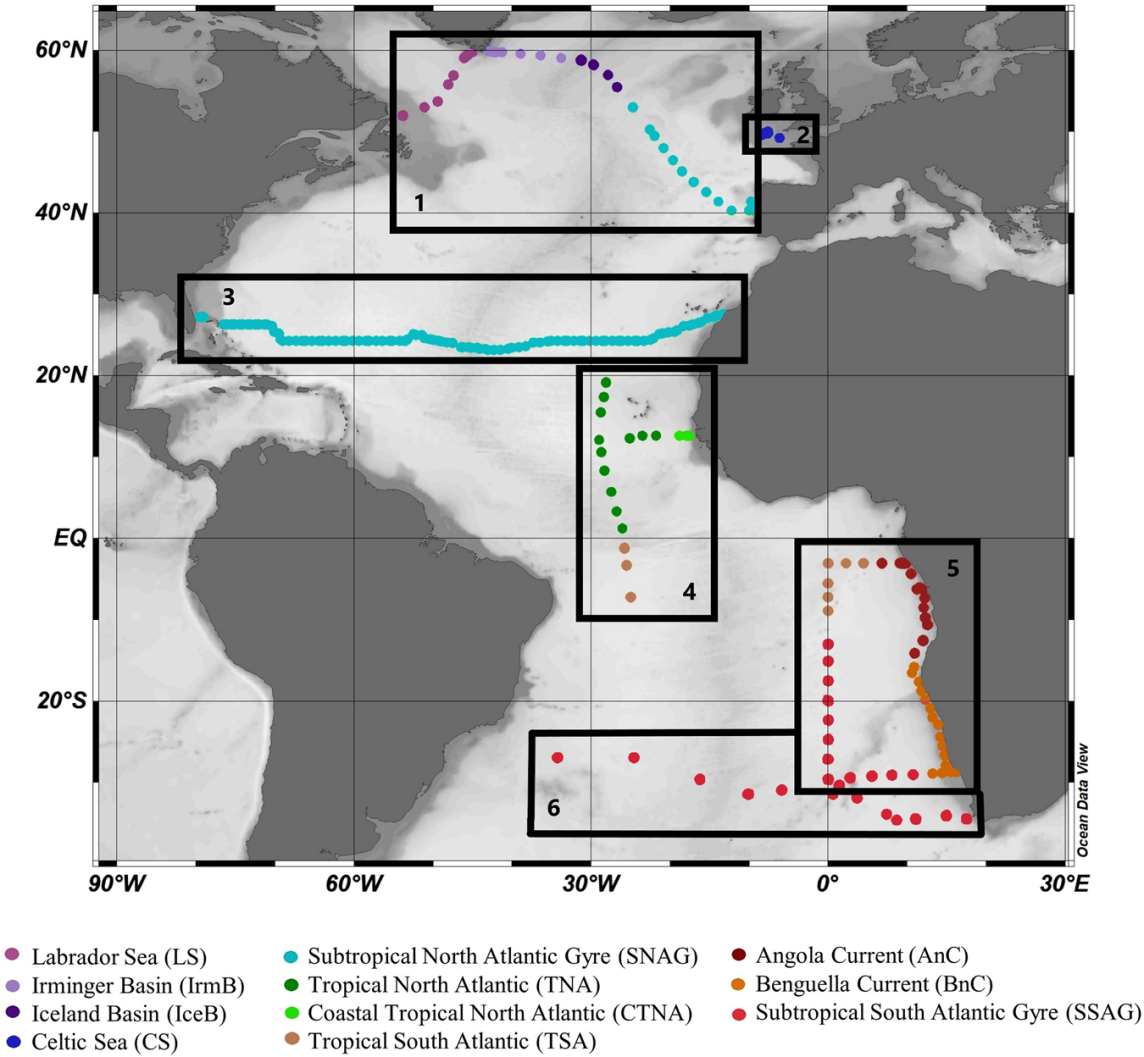
Sampling map of research cruises from 2005 to 2015 included in this study, listed here geographically from north to south. Boxes mark the broader region and track of each research cruise; (1) GEOVIDE cruise (GEOTRACES – GA01 section) in May-April 2014, (2) CD173 in July-August 2005, (3) D346 in January-February 2010, (4) D361 (GEOTRACES – GA06 section) in February-March 2011, (5) M121 (GEOTRACES – GA08 section) in December 2015, and (6) M124 in February-March 2016. Dots indicate the sampled stations. Colours indicate the grouping of data in oceanographic regions listed here geographically from North to South; Labrador Sea (LS), Irminger Basin (IrmB), Iceland Basin (IceB), Celtic Sea (CS), Subtropical North Atlantic Gyre (SNAG), Tropical North Atlantic (TNA), Coastal Tropical North Atlantic (CTNA), Tropical South Atlantic (TSA), Angola Current (AnC), Benguela Current (BnC) and Subtropical South Atlantic Gyre (SSAG). The figure was produced using Ocean Data View^99^ v.4.7.9 (Schlitzer, R. Ocean Data View. 2018, https://odv.awi.de/).

We compiled newly analysed (unpublished) and previously published data from the Atlantic Ocean collected over a time span from 2005 to 2016. We present here new data (total of 306 data points for heme *b*) from the research cruises M121 (2015) and M124 (2016) in the South Atlantic. We further obtained published data (total of 668 data points for heme *b*) for the research cruises CD173^16^, D346^15^, D361^50^ and GEOVIDE^36^. Details on the expeditions are listed in Table 1 in chronological order and a map of the sampling areas and stations is shown in Fig. 1. These research expeditions and thus datasets cover several biogeochemical provinces of the Atlantic Ocean. For statistical analysis, data interpretation and discussion, we grouped the sampled stations into sub-regions according to the geographical location and the chlorophyll *a* (chl *a*) distribution. Here we list these regions in geographical order (north to south); Labrador Sea, Irminger Basin, Iceland Basin, Celtic Sea, Subtropical North Atlantic Gyre, Tropical North Atlantic, Coastal Tropical North Atlantic, Tropical South Atlantic, Angola Current, Subtropical South Atlantic Gyre and Benguela Current.

**Table 1:** List of the research cruises in the Atlantic Ocean included in this study and number of total heme *b* data points (i.e. both above and below the mixed layer depth) per cruise.

Cruise ID	Oceanographic Area	Year	Month(s)	Research Vessel	Heme *b* data points	References
CD173	Celtic Sea	2005	July-August	RRS Charles Darwin	29	^16^
D346	Subtropical North Atlantic Gyre	2010	January-February	RRS Discovery	379	^15^
D361	Tropical North Atlantic Tropical South Atlantic	2011	February-March	RRS Discovery	89	^50^
GEOVIDE	Subpolar and Subtropical North Atlantic Ocean	2014	May-June	RV Pourquoi Pas?	171	^36^
M121	(Sub)-Tropical South Atlantic Benguela Upwelling Angola Dome – Congo Plume	2015	December	FS Meteor	254	This study
M124	Subtropical South Atlantic	2016	February-March	FS Meteor	52	This study

References indicate previous studies where heme *b* data were published.

### Particulate organic carbon and chlorophyll *a*

Particulate Organic Carbon (POC) concentrations ranged from 0.14 to 63.5 μmol L^−1^ (median = 3.1 μmol L^−1^, n = 1137) for all oceanographic regions from the surface down to 220 m depth. The highest concentrations of POC were determined in the high latitude North Atlantic Ocean (Labrador Sea, Irminger Basin, Iceland Basin; median = 5.6 μmol L^−1^, n = 203) and the coastal areas (Celtic Sea, Coastal Tropical North Atlantic, Angola Current, Benguela Current; median = 8.0 μmol L^−1^, n = 195). Concentrations of POC were lowest in both subtropical gyres (North Atlantic Gyre, South Atlantic Gyre) and open ocean stations of the Tropical North and South Atlantic with a median value of 2.4 μmol L^−1^ (n = 752).

Chlorophyll *a* concentrations ranged from <0.01 to 10.7 nmol L^−1^ (median = 2.0 nmol L^−1^, n = 1272) overall (0–220 m depth). Similar to POC, chl *a* was highest in the subpolar North Atlantic (Labrador Sea, Irminger Basin, Iceland Basin; median = 0.50 nmol L^−1^, n = 186) and the coastal areas (Celtic Sea, Coastal Tropical North Atlantic, Angola Current, Benguela Current; median = 0.38 nmol L^−1^, n = 311). Lower concentrations of chl *a* were determined in tropical areas (Tropical North Atlantic, Tropical South Atlantic; median = 0.21 nmol L^−1^, n = 128) and in the subtropical gyres (North Atlantic Gyre, South Atlantic Gyre; median = 0.13 nmol L^−1^, n = 66). The lowest chl *a* values were observed in the Subtropical South Atlantic Gyre, where we determined a median concentration of 0.03 nmol L^−1^. Our results are comparable to those reported during the Atlantic Meridional Transect (AMT) cruises in this region^51–53^.

**Figure 2:**
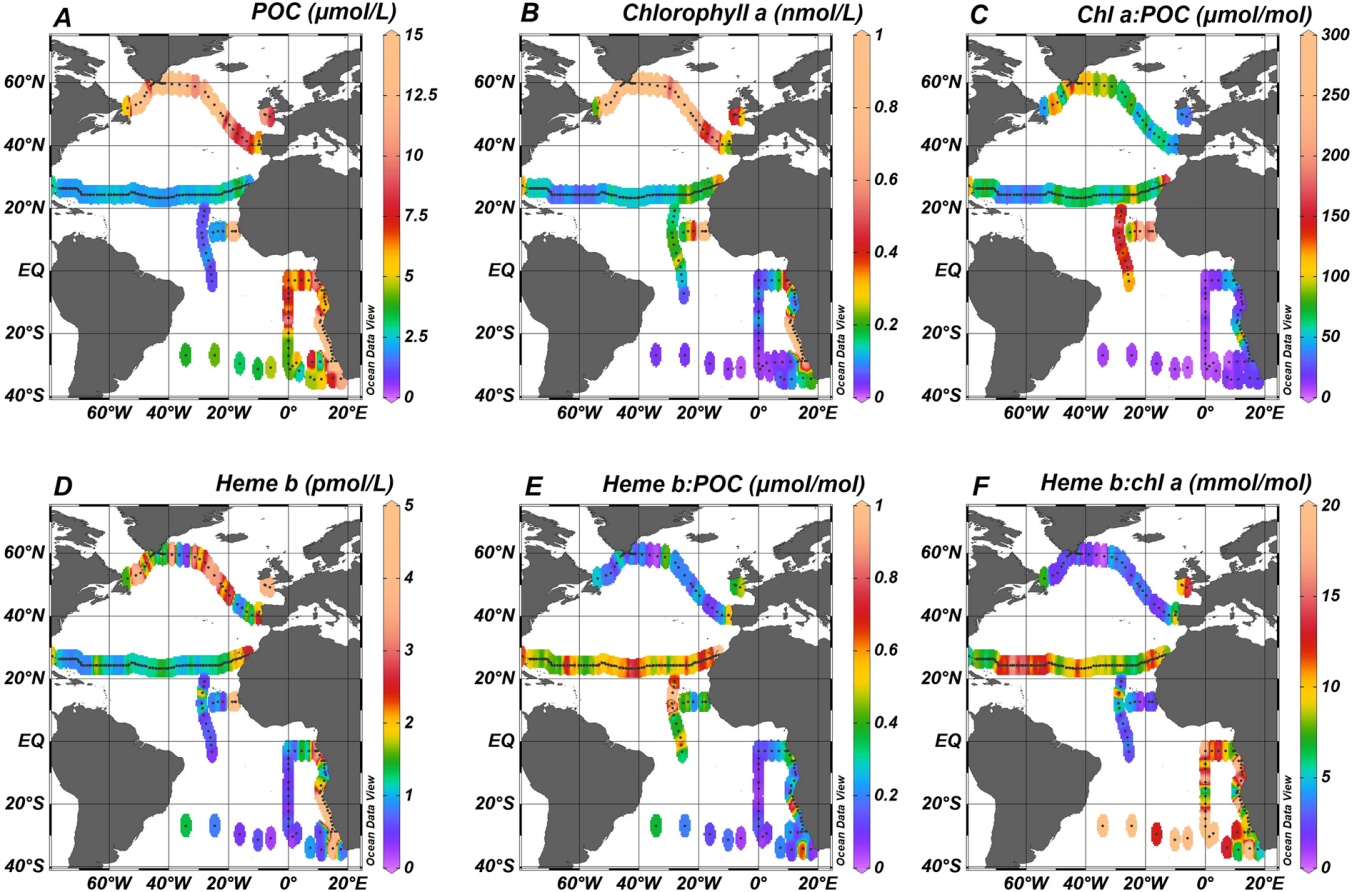
Spatial distribution of (**A**) Particulate organic carbon (POC), (**B**) chlorophyll *a* (chl *a*), (**C**) heme *b*, (**D**) heme *b*:POC, (**E**) heme *b*:chl *a*, and (**F**) chl *a*:POC. Values represent median the values calculated for the Surface Mixed Layer (SML). Dots indicate the sampled stations. The figure was produced using Ocean Data View^99^ v.4.7.9 (Schlitzer, R. Ocean Data View. 2018, https://odv.awi.de/).

**Figure 3:**
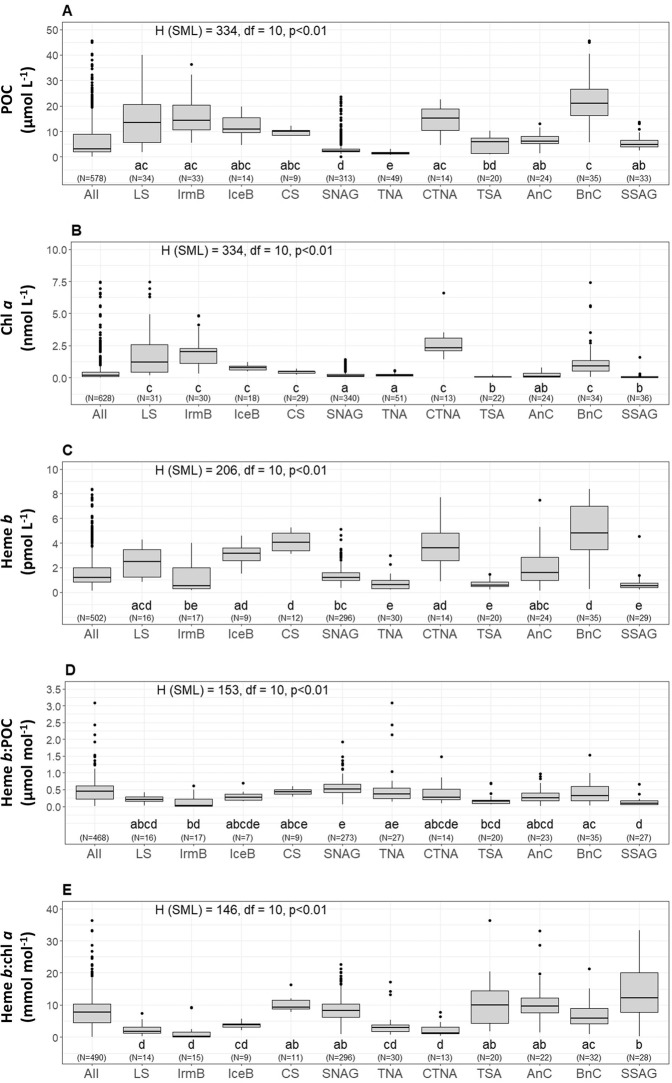
Boxplots of distributions in the surface mixed layer (SML) of (**A**) Particulate Organic Carbon (POC), (**B**) chlorophyll *a* (chl *a*), (**C**) heme *b*, (**D**) the ratio heme *b*:POC, and (**E**) the ratio heme *b*:chl *a* for the eleven oceanographic regions; Labrador Sea (LS), Labrador Sea (LS), Irminger Basin (IrmB), Iceland Basin (IceB), Celtic Sea (CS), Subtropical North Atlantic Gyre (SNAG), Tropical North Atlantic (TNA), Coastal Tropical North Atlantic (CTNA), Tropical South Atlantic (TSA), Angola Current (AnC), Benguella Current (BnC) and Subtropical South Atlantic Gyre (SSAG). The boxes show the interquartile range (IQR) of values. The bold lines inside the boxes indicate the median value for each region and divide the boxes the 25th (bottom) and 75th (upper) percentiles. The upper whisker represents the largest value no farther than 1.5 times the IQR, and the bottom whisker indicates the smallest value no farther than 1.5 times the IQR. Dots represent the outliers of the distributions. On top of sub-plots A, B, C, D and E the result of the Kruskal-Wallis test is annotated. Letters (letters a to d) below the boxes indicate the Compact Letter Display (cld) of the statistically significant different groups in the SML after a Post-hoc test for multiple comparisons of groups. The numbers in brackets indicate the number of data points used per box for each parameter. The figure was produced using R Statistical Software^97^ v.1.0.136 (R core team, 2016, https://www.R-project.org).

Depth profiles (Supplementary Fig. S1) of POC and chl *a* indicated generally higher concentration within the surface mixed layer (SML) that decreased with depth (Wilcoxon Rank Sum test, *p* < *0.01*). However, clear Deep Chlorophyll Maxima (DCM) were also present in most of the oceanographic regions (i.e. Labrador Sea, Celtic Sea, (sub-)tropical North Atlantic, Angola Current, Benguela Current and (sub-)tropical South Atlantic) (Supplementary Fig. S1). A summary of the median concentrations and the ranges of POC and chl *a* in the SML is reported on Table 2. Plots A and B in Fig. 2 illustrate the median concentrations of POC and chl *a* respectively for each sampling station across the Atlantic Ocean. Chlorophyll *a* correlated with POC (Spearman’s rho, r = 0.57, n = 556, p < 0.01) in the SML (Supplementary Fig. S2A). The Kruskal-Wallis test confirmed statistically significant differences among the oceanographic regions (H = 334, p < 0.01) (Fig. 3A,B).

**Table 2:** Statistics summary table of heme *b*, chlorophyll *a* (chl *a*), particulate organic carbon (POC), heme *b*:POC and heme *b*:chl *a* in the eleven oceanographic regions in the Atlantic Ocean from 2005 to 2015.

Region	Stats	Heme *b*	Chl *a*	POC	heme *b*:POC	Heme *b*:chl *a*
		(pmol L^−1^)	(nmol L^−1^)	(μmol L^−1^)	(μmol mol^−1^)	(mol mol^−1^)
Labrador Sea	Median	2.5	1.2	13.5	0.20	1.9
	Range	0.82 ± 4.3	0.20 ± 7.4	1.7 ± 40.0	0.03 ± 0.41	0.18 ± 7.5
	No. samples	(16)	(31)	(34)	(16)	(14)
Irminger Basin	Median	0.53	2.0	14.3	0.03	0.20
	Range	0.16 ± 4.0	0.32 ± 4.8	5.4 ± 36.4	0.01 ± 0.61	0.07 ± 9.4
	No. samples	(17)	(30)	(33)	(17)	(15)
Iceland Basin	Median	3.2	0.77	10.9	0.28	3.8
	Range	1.5 ± 4.6	0.47 ± 1.2	4.6 ± 19.7	0.16 ± 0.70	2.2 ± 5.7
	No. samples	(9)	(18)	(14)	(7)	(9)
Celtic Sea	Median	4.1	0.47	10.1	0.43	9.32
	Range	3.1 ± 5.3	0.21 ± 0.72	8.2 ± 12.2	0.28 ± 0.60	7.8 ± 16.3
	No. samples	(12)	(29)	(9)	(9)	(11)
Subtropical North Atlantic Gyre	Median	1.2	0.15	2.3	0.52	8.3
	Range	0.30 ± 5.1	0.05 ± 1.4	0.16 ± 23.5	0.06 ± 6.2	0.88 ± 22.7
	No. samples	(296)	(340)	(313)	(273)	(296)
Tropical North Atlantic	Median	0.6	0.21	1.3	0.37	2.9
	Range	0.23 ± 3.0	0.10 ± 0.57	0.73 ± 3.0	0.13 ± 3.1	0.77 ± 79.6
	No. samples	(30)	(51)	(49)	(27)	(30)
Coastal Tropical North Atlantic	Median	4.2	2.3	15.2	0.27	1.4
	Range	0.89 ± 15.7	1.42 ± 6.6	4.6 ± 22.6	0.09 ± 1.49	0.47 ± 7.8
	No. samples	(14)	(13)	(14)	(14)	(13)
Tropical South Atlantic	Median	0.55	0.08	6.0	0.16	10.0
	Range	0.22 ± 1.5	0.02 ± 0.23	1.0 ± 10.2	0.05 ± 0.70	1.8 ± 36.3
	No. samples	(20)	(22)	(20)	(20)	(20)
Angola Current	Median	1.6	0.12	6.2	0.25	9.7
	Range	1.60 ± 7.5	0.01 ± 0.79	1.4 ± 13.1	0.01 ± 0.98	1.5 ± 215
	No. samples	(24)	(24)	(24)	(23)	(22)
Benguela Current	Median	6.2	0.91	22.0	0.31	6.0
	Range	0.25 ± 33.7	0.05 ± 7.4	5.6 ± 63.5	0.02 ± 1.5	0.9 ± 138
	No. samples	(35)	(34)	(35)	(35)	(32)
Subtropical South Atlantic Gyre	Median	0.51	0.03	4.9	0.09	12.7
	Range	0.20 ± 4.5	0.01 ± 1.6	2.4 ± 13.6	0.04 ± 0.67	0.32 ± 45.6
	No. samples	(29)	(36)	(33)	(27)	(28)

The ratio of chl *a*:POC ranged from 0.21 to 8286 μmol mol^−1^ (SML_median_ = 67.3 μmol mol^−1^, n = 550: DCM_median_ = 40.6 μmol mol^−1^, n = 520) for the whole dataset (Fig. 2C). The highest values in the SML were determined in the Coastal Tropical North Atlantic (median = 189 μmol mol^−1^, n = 13) and the Tropical North Atlantic (median = 155 μmol mol^−1^, n = 48) whilst chl *a*:POC was lowest in the subtropical South Atlantic Gyre (median = 7.62 μmol mol^−1^, n = 32). Statistically significant differences were determined between the SML and the DCM (Wilcoxon Rank Sum test, *p* < *0.01*) and between oceanographic regions (SML: H = 253.27, df  = 10, *p* < 0.01) which were mainly attributed to the differences between the North (sub-) tropical Atlantic (i.e. Subtropical North Atlantic Gyre, Tropical North Atlantic and Coastal Tropical North Atlantic) and the South Atlantic (Subtropical South Atlantic Gyre). Large differences between chl *a*:POC ratios in the North And South Atlantic have also been observed previously and are thought to be strongly influenced by phytoplankton physiology^53^.

### Heme *b* concentrations and ratios

Heme *b* concentrations ranged from 0.10 to 33.7 pmol L^−1^ (median = 1.16 pmol L^−1^, n = 974) from the surface layer down to 200 m depth. The depth profiles of heme *b* (Supplementary Fig. S1) indicated that heme *b* was typically higher in the SML (SML: median = 1.23 pmol L^−1^, n = 502) and decreased with depth (Wilcoxon rank sum test, p < 0.01) except for the cases where a DCM was present (Labrador Sea, Celtic Sea and Benguela Current).

**Figure 4:**
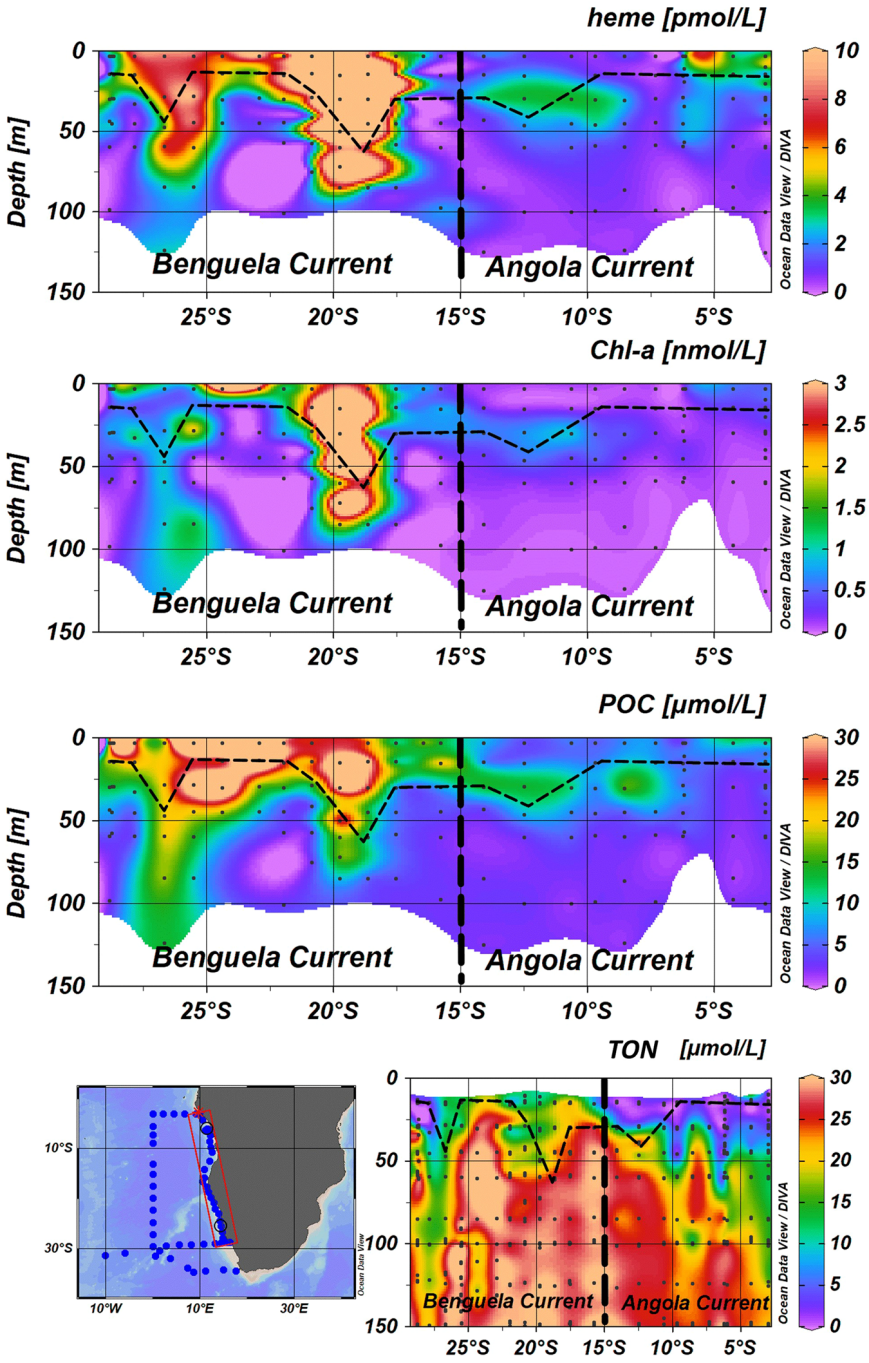
Section plots of heme *b*, chlorophyll *a* (chl *a*), particulate organic carbon (POC) and total oxidized nitrogen (TON – sum of nitrite and nitrate) along the coast of Africa during the M121 cruise (December 2015). The contour plots correspond to the stations closest to the coast (13–17 °E). Dashed lines indicate the Mixed Layer Depth (MLD). Dots indicate the sampled stations and the data points available. Letter annotations indicate the two oceanographic regions encountered which were the Benguela Current and Angola Current regions. The figure was produced using Ocean Data View^99^ v.4.7.9 (Schlitzer, R. Ocean Data View. 2018, https://odv.awi.de/).

The median concentration of heme *b* in the SML is shown in Fig. 2D. Statistically significant differences were observed in the spatial distribution of heme *b* in the SML for the various oceanographic regions (Kruskal-Wallis, H = 206, p < 0.01) (Fig. 3C). Significantly higher concentrations were observed in the SML of the coastal regions (Celtic Sea, Coastal Tropical North Atlantic, Angola Current, Benguela Current), ranging from 0.10 to 33.7 pmol L^−1^ (median = 4.1 pmol L^−1^, n = 85). These regions include two upwelling areas (Coastal Tropical North Atlantic and Benguela Current) and the Congo river plume located in the Angola region; hence, heme *b* was highest in the Benguela area (median = 6.2 nmol L^−1^, n = 14) (Fig. 4) followed by the Coastal Tropical North Atlantic (median = 4.2 nmol L^−1^, n = 14) (Table 2). In addition, heme *b* was also enhanced in the highly productive subpolar areas (Iceland Basin, Greenland Shelf and Labrador Sea) which were sampled during the spring bloom in 2014 (range 0.16–4.6 pmol L^−1^, median = 2.2 pmol L^−1^, n = 48)^36^. However, the Irminger Basin deviated from this pattern, despite being sampled in the same season, exhibiting the lowest heme *b* concentrations (median = 0.53 pmol L^−1^, n = 17)^36^, along with the Subtropical South Atlantic Gyre (median = 0.51 pmol L^−1^, n = 29) and the offshore stations in the tropical Atlantic (Tropical South Atlantic and Tropical North Atlantic, median = 0.58 pmol L^−1^, n = 50).

Heme *b* correlated with both POC (Spearman’s rho, r = 0.41, n = 465, p < 0.01) and chl *a* (Spearman’s rho, r = 0.56, n = 490, p < 0.01) (Supplementary Fig. S2B,C) in the SML. These results along with the average depth profiles of heme *b*, chl *a* and POC (Supplementary Fig. S1) suggest that both the spatial and the vertical distribution of heme *b* concentrations in the Atlantic Ocean were strongly influenced by biomass, as observed previously^15^. However, heme *b* was shown in previous studies to vary due to phytoplankton regulation strategies that occur as adaptation responses to iron-limitation^16,36^, thus weakening the overall correlation of heme *b* relative to POC.

In order to examine the changes of heme *b* relative to biomass as indicated by POC and chl *a*, we calculated the ratios of heme *b*:POC and heme *b*: chl *a*^16,35^. The heme *b*:POC ratio represents the heme *b* per unit carbon derived from the total particulate organic matter and previous field studies showed that this ratio varied as a result of iron availability^15,16,35,36^. The ratio heme *b*: chl *a* corresponds to organic matter originating from phytoplankton, but because of intracellular chlorophyll variability, can also be influenced by light- and nutrient-driven changes in the photosystem.

In this study, heme *b*: POC ranged overall from 0.01 to 6.2 μmol mol^−1^ (median = 0.39 μmol mol^−1^, n = 907). However, the difference in values between the two layers (above and below the SML) was statistically significant (Wilcoxon Rank Sum test, p < 0.01), with heme *b*: POC significantly higher in the DCM. The depth profiles (Supplementary Fig. S1) showed that the increase in heme *b*: POC in the DCMs was accompanied by an increase in the chl *a*:POC suggesting photoacclimation of phytoplankton to low light and higher investment in the photosynthetic proteins^15,54,55^. Statistically significant differences were observed for the SML for the various oceanographic regions in heme *b*:POC (Kruskal-Wallis, H = 153, p < 0.01) (Fig. 3D). The lowest values of heme *b*:POC were determined in the Irminger Basin (SML: median = 0.03 μmol mol^−1^, n = 17) and the Subtropical South Atlantic Gyre (SML: median = 0.08 μmol mol^−1^, n = 27) suggesting a decoupling of heme *b* from POC in these regions (Fig. 2E). Similarly, we observed statistically significant differences among the oceanographic regions for the heme *b*:chl *a* ratio (Kruskal-Wallis, H = 146, p < 0.01) (Fig. 3E). Heme *b*:chl *a* ratios ranged overall from 0.07 to 327 mmol mol^−1^ (median = 7.3 mmol mol^−1^, n = 942) and followed a different pattern of spatial distribution in the SML compared to heme *b*:POC (Fig. 2F). Hence, heme *b*: chl *a* was lowest in the SML of the Irminger Basin (SML: median = 0.20 mmol mol^−1^, n = 15) and highest in the Subtropical South Atlantic Gyre (SML: median = 12.7 mmol mol^−1^, n = 28) suggesting community driven differences in photoacclimation and/or hemoprotein regulation patterns.

### Identification of heme *b* based criteria for iron limited phytoplankton

In laboratory studies, values of heme *b*:POC below 0.10 μmol mol^−1^ were indicative of iron-limited phytoplankton^15,16^, and similar values have been determined for field phytoplankton communities in the iron - limited post-bloom Iceland Basin^35^ and low-iron Southern Ocean^16,35^. In case of Irminger Basin, Louropoulou *et al*.^36^ showed heme *b* depletion (median = 0.53 pmol L^−1^, n = 17) in a large diatom-dominated community in May-June 2014 despite relatively high iron concentrations (≥0.30 nmol L^−1^), indicating iron limitation resulting from the high iron requirements of the extant phytoplankton population.

In this study, we report a similar pattern of low heme *b* concentrations (<1 pmol L^−1^) and low heme *b*:POC ratios (<0.1 μmol mol^−1^) in the eastern and in the central Subtropical South Atlantic Gyre, which point to iron-limited phytoplankton communities in these areas. Our observations are confirmed by bioassay experiments that showed the eastern boundary of the Subtropical South Atlantic Gyre was nitrate-iron co-limited during the period of the cruise^45^. The good agreement between the heme *b* measurements and the bioassay experiments of Browning, *et al*.^45^ reinforces previous comparisons with other approaches for mapping iron-limited phytoplankton communities^36^ that included the dissolved iron:nitrate ratio^56^, modified Si^*^ tracer^57–59^ and satellite-derived quantum yield of fluorescence Φ_sat_^60,61^. Heme *b* measurements thus appeared to successfully map iron limited phytoplankton by depicting the momentary condition of the phytoplankton cells *in situ*.

We constructed a histogram of all our SML heme *b*:POC data in order to investigate overall trends in Atlantic Ocean phytoplankton populations. The distribution of the heme *b*:POC data (Supplementary Fig. S3A) was skewed to the right with a median value of 0.44 μmol mol^−1^ (n = 468) and a mean value of 0.47 ± 0.42 μmol mol^−1^ (n = 468). Furthermore, both the Irminger Basin and the Subtropical South Atlantic Gyre exhibited an exponential distribution and their medians (0.03 μmol mol^−1^ and 0.09 μmol mol^−1^ respectively) deviated from the median of the distribution (Supplementary Fig. S3A). Taking into account laboratory studies and field observations of heme *b*: POC ratios and their relationship to iron limitation, we suggest that 0.10 µmol mol^−1^ is a reasonable estimate for defining an anemic, iron limited phytoplankton community in the Atlantic Ocean. Hence, the data appearing in the left tail (break 0.10 μmol mol^−1^, n = 50) (Supplementary Fig. S3A) of the distribution point to iron limited phytoplankton communities. Thus, by this definition, the southern stations of Tropical South Atlantic were iron limited and iron (co-)limitation is predicted to extend to the central parts of the subtropical gyre (Fig. 2, cruise M124, sampling February-March 2016).

In contrast, the western boundaries of the Subtropical South Atlantic Gyre were characterized by slightly higher heme concentrations (1.36 pmol L^−1^) and heme *b*:POC ratios (0.37 μmol mol^−1^) compared to the eastern and central Subtropical South Atlantic Gyre. These results suggest that this area was not iron limited; indeed Rijkenberg, *et al*.^38^ reported iron concentrations up to 6.1 nM in the upper 800 m off shore of Brazil in the Subtropical Shelf Front (STSF), which is formed by the southward flowing Brazil Current and the norward flowing Malvinas Current^62^. The iron enrichment was attributed to aeolian deposition and transport by the STSF, and to offshore export of iron from Brazilian shelf waters and the Rio de la Plata river^38,62^.

The heme *b*:POC values of the open ocean stations in the Tropical North Atlantic, (SML; median = 0.26 μmol mol^−1^) do not point towards iron-limited phytoplankton communities, even though heme *b* concentrations were low (SML; median = 0.59 pmol L^−1^); we attribute this trend to low biomass at the time of sampling. In general, the location of the Intertropical Convergence Zone (ITCZ) defines the dust derived iron supply to surface waters^49^ and thus influences the biogeochemical status and bloom progression between the northern and the southern oligotrophic waters around the Equator^63,64^. In February-March 2011, when sampling was performed, Schlosser, *et al*.^63^ reported that the ITCZ was at ∼1°N and the Tropical North Atlantic was receiving significant amounts of atmospheric deposition whilst the Tropical South Atlantic had low atmospheric dust concentrations. In addition, Snow, *et al*.^64^ reported increased nitrogen fixation rates due to the abundance of *Trichodesmium sp*. between 15°N and 7°S which have high iron requirements relative to non-nitrogen fixing phytoplankton.

### Heme *b* regulation strategies?

Similar to the heme *b*:POC ratio, the distribution of the population for the heme *b*:chl *a* ratio (Supplementary Fig. S3B) was also skewed to the right with median value of 7.77 mmol mol^−1^ and a mean value 8.98 mmol mol^−1^ (n = 491). However, we observed a contrasting behaviour in the ratios of the two iron limited areas Irminger Basin (median_SML_ = 0.20 mmol mol^−1^, n = 20) and Subtropical South Atlantic Gyre (median_SML_ = 12.7 mmol mol^−1^, n = 28), despite the similar trend in heme *b*:POC. The medians of heme *b*:chl *a* for these two areas deviated from the overall median of the distribution and located either on left (Irminger Basin) or the right (Subtropical South Atlantic Gyre) tail. Heme *b* exhibited the same trend for the two areas which implies that changes in chl *a* quotas drove the differences in the ratio. Hence, we ascribed this contrast to different extant phytoplankton groups, to different photoacclimation and nutrient status.

The low heme *b*:chl *a* observed in Irminger Basin implied that the heme *b* containing proteins of the photosynthetic apparatus decreased whilst chl *a* was conserved. In culture, several diatoms and prymnesiophytes exhibited decreased heme *b*:chl *a* ratio under low iron conditions (<0.50 nmol L^−1^)^15,16^ implying allocation of iron away from the hemoprotein pool^16^. This behaviour was considered an adaptation strategy of phytoplankton that would allow a reduction of the overall iron requirements and a more efficient utilization of the available iron in order to sustain growth^16^. Here, this pattern was also observed in the field in the Irminger Basin (GEOVIDE, May-June 2014) for diatom populations that likely employed heme *b* regulation^36^ in order to adapt to declining iron concentrations during bloom progression. For example, allocating the iron away from the hemoproteins would lead to decline of the heme *b*-containing cytochromes *b*_6_*f* and *b*_559_ of the PSII apparatus^8,18,28^, which in turn would be accompanied by increases in the chl *a*:PSII ratios. Indeed this was observed by Macey, *et al*.^65^ in the post-bloom iron-limited Iceland Basin. Particularly for the eukaryotes, another strategy of reducing iron requirements is switching from nitrate to ammonium utilization^66–68^ which induces the reduction of heme *b*-containing nitrate reductase.

We observed very high heme *b*:chl *a* in the Subtropical South Atlantic Gyre which was driven by low concentrations of chl *a* in the area (SML median = 0.03 nmol L^−1^). Furthermore, the ratio chl *a*:POC was 7.68 μmol mol^−1^ (n = 32) in that region pointing to lower photoacclimation of the dominant phytoplankton groups. At the time of the study, haptophytes and the picophytoplankton species *Synechococcus* and *Prochlorococcus* were most dominant in the eastern parts of the gyre^45^. Similar community composition characterizes the Subtropical North Atlantic Gyre^69^. Therefore, we made a comparison of the trends in chl *a*:POC between these two regions; in the Subtropical North Atlantic Gyre, the chl *a*:POC was higher compared to the Subtropical South Atlantic Gyre reaching a median value of 65.0 μmol mol^−1^ (n = 312). The contrasting behaviour in chl *a*:POC can be ascribed to differences in light climate, which is known to strongly influence chl *a*:POC ratios^70^. Higher light conditions in the Subtropical South Atlantic Gyre in comparison to the Subtropical North Atlantic Gyre, as a result of both higher incident irradiance during winter months^71^ and shallower mixed layers (Supplementary Fig. S1), would be expected to result in the lower chlorophyll-to-carbon ratios observed. However, the low chl *a* concentrations, and thus the low chl *a*:POC values, could also be a result of general nutrient limitation in the area as shown previously^72,73^. Hence, our results implied a high variability in chl *a* due to a combination of photoacclimation, nutrient limitation, phytoplankton physiology and species specific modifications in the photosynthetic apparatus.

Our heme *b* data from the field appear to be broadly consistent with laboratory studies in terms of magnitude and relationship with POC. Nevertheless, a key challenge in the interpretation of relationships between heme *b*, chl *a* and POC is distinguishing between variability resulting from physiological responses to changes in light and nutrient regimes, and variability resulting from changes in the relative contributions of different carbon (C) pools^35^. The relationship between biomass and POC is also critical to the interpretation of optical parameters measured from space. Studies of interrelationships between chl *a*, POC and phytoplankton biomass in the Atlantic Ocean have concluded that chl *a*:POC ratios vary to a greater extent than C biomass:POC^70,74^, with the greater variability in chl *a*:POC attributed to changes in phytoplankton photophysiology driven by nutrient and light regimes. Thus in analogy, we have assumed throughout our discussion that variability between heme *b*, chl *a* and POC reported here will be more strongly influenced by physiological changes than by changes in POC composition. Further support for this assumption, or alternative methods of assessing phytoplankton biomass would be useful in future applications of heme *b* as an indicator of iron status in the field.

### Comparison of field heme *b* concentrations with model-based predictions

**Figure 5:**
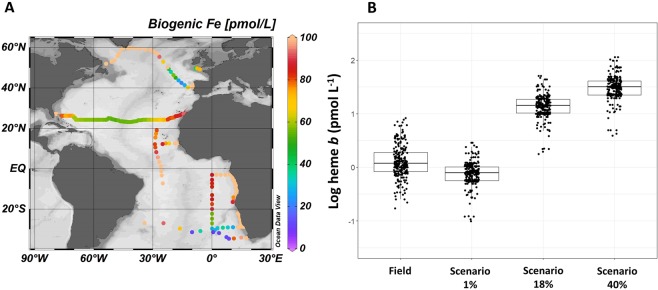
(**A**) Model-based estimation the biogenic iron (Fe_bio_) pool deriving from the sum of the nanophytoplankton and diatom iron pools in pmol L^−1^. Colours represent the monthly mean values for the upper most layer of the model calculated for the same time as sampling (depth 10 m). (**B**) Actual field heme *b* measurements and predicted heme *b* concentrations calculated from the total Fe_bio_ concentrations in the surface mixed layer (SML) of each station. Calculations follow three scenarios (1%, 18%, and 40%) representing the heme *b* proportion relative to the total Fe_bio_ according to observations in cultured phytoplankton^15^. Plot A was produced using Ocean Data View^99^ v.4.7.9 (Schlitzer, R. 2018, https://odv.awi.de/). Plot B was produced using R statistical software^97^ v.1.0.136 (R core team, 2016, https://www.R-project.org).

Both theoretical estimates and direct measurements suggest heme *b* represents an important amount of the total cellular iron pool in phytoplankton (mean 18 ± 14%)^14,15,75^ thus the second part of our study aimed to examine to what extent the heme *b* field data were comparable to heme *b* estimated from the biogenic iron pool as predicted from a global biogeochemical model. Here we used the PISCES-v2 (Pelagic Interactions Scheme for Carbon and Ecosystem Studies volume 2) biogeochemical model to estimate the biogenic iron pool which is determined based on Michaelis Menten uptake kinetics, further regulated by a maximum iron-to-carbon (Fe:C) cellular quota and enhanced iron uptake under iron limitation^76–78^. Figure 5A illustrates the biogenic iron (Fe_bio_) pool from the model which corresponds to sum of the diatom and nanophytoplankton iron pools extracted at the same geographic position and month as the field data. Values represent the monthly means for the upper most layer of the model (depth 10 m) and ranged from 9.20 to 285 pmol L^−1^ (median = 78.8 pmol L^−1^, n = 254). Although a direct comparison of the absolute values is questionable due to the large uncertainties associated with model predictions of the ocean iron cycle^79^, there are similar trends between the Fe_bio_ and heme *b* abundance in the Atlantic Ocean. In particular, both parameters were highest in the subpolar North Atlantic (Labrador Sea, Iceland Basin), along the continental margins (Celtic Sea, Angola Current) and in the upwelling areas (Coastal Tropical North Atlantic, Benguela Current), whilst the lowest values were determined in the oligotrophic Subtropical South Atlantic Gyre.

According to phytoplankton culture experiments with different phytoplankton species, heme *b* accounts for between 1 and 40% of the particulate Fe (PFe) (averaging 18 ± 14%)^14,15,80^. We calculated the predicted heme *b* concentrations (in pmol L^−1^) from the modelled Fe_bio_ of each station in the SML using three scenarios (values) 1%, 18% and 40% that represent the range and mean of heme *b*:PFe observed in laboratory cultures. We used the formula heme *b* = [Fe_bio_ * [heme *b* ⁄ PFe] _lab_]/100, where [heme *b* / PFe]_lab_ denotes the proportion of heme *b* relative to PFe determined for cultured phytoplankton under various growing conditions.

Predicted heme *b* concentrations were lowest for the 1% scenario and highest for the 40% scenario (Fig. 5B) with the 1% scenario corresponding more closely to the field observations reported in this study. This trend was further supported by the ratio heme *b*:Fe_bio_ calculated from the mean heme *b* concentrations (in the SML) and the model-based Fe_bio_ which ranged from 0.17% to 9.1% (median = 1.8%, n = 234). Taken together with heme *b*:POC, these results suggest that, as far as heme *b* is concerned, field populations were comparable to laboratory observations for species with low heme *b* content. Furthermore, the ratio of 1% was determined in cultures where iron (0.50 nmol L^−1^) or nitrate supply had been exhausted, thus perhaps better representing the natural environment. Therefore, the model prediction of Fe_bio_ and the *in situ* heme *b* concentrations were consistent with previous laboratory observations^15,16^. The differences in the proportion of heme *b* relative to Fe_bio_ likely arose because of the microbial community composition of each region and the interspecific differences in hemoprotein processes.

Biogenic iron and iron-to-carbon (Fe:C) ratios are critical for linking the iron and carbon cycles in the ocean and constraining phytoplankton iron requirements within biogeochemical models^81^. Currently, the biogenic iron pool can be assessed by radioisotope uptake experiments^81,82^, single-cell synchrotron x-ray fluorescence^81,83,84^ and determination of particulate iron after careful removal of or correction for lithogenic iron^81,85–87^. Whilst all these techniques provide complimentary and useful information on particulate iron, single cell iron-to-phosphorus ratios or community wide Fe:C uptake rates, none provide a definitive estimate of average *in-situ* biogenic iron quotas within phytoplankton populations. Furthermore, none of the above methods were applied consistently on larger scale field expeditions. The good agreement of heme *b* observational and model-based data suggests that heme *b* could potentially serve as an indicator of the biogenic iron for field studies and provide an assessment of the proportion of iron used in the heme *b* and the hemoprotein pools in marine phytoplankton, although further comparison of heme *b* abundance with established methods of assessing biogenic iron are required.

## Conclusions

In this study, we suggest that the ratio heme *b*:POC can serve as a reliable indicator of iron limitation since it appeared not to be influenced by species- specific processes which potentially involve modifications in the hemoprotein and/or other protein pools. A comparison of the heme *b* method with already established protocols, such as bioassay experiments^43,56,88^ and iron-stress biomarkers (e.g. flavodoxin, ferredoxin and IdiA)^25,31,34,89,90^, would validate this simple method for mapping iron limited phytoplankton in small or large scale field expeditions. Although determination of heme *b* in marine particulate material requires specialist analytical instruments, sample collection is straightforward and utilises the same approaches routinely applied for collection of pigment samples. Importantly, sampling for heme *b* does not require trace metal clean conditions, significantly widening the potential for assessment of iron limitation in the field. Furthermore, heme *b* is a ubiquitous biomolecule and is thus not dependent on the presence of particular phytoplankton species.

The variability of the heme *b* to biomass ratios suggested that phytoplankton 1) employ diverse mechanisms to utilize the available iron in the hemoprotein pool, and 2) are able to regulate the hemoproteins under iron limiting conditions. Expanding the field and laboratory research in hemoprotein abundance and cycling would contribute to the identification of such iron utilization and regulation strategies. One aspect could be the transcriptomic and proteomic analyses of biomolecules involved in the heme *b* cycling pathways. These include the proteins associated with the utilization of the heme *b* cofactor, such as the components of the photosynthetic or nitrate assimilation apparatus, as well as the components of the biosynthesis and breakdown pathways. The information about iron-utilization in hemoproteins and about regulation of the hemoprotein pool would shed light on the molecular response and adaptation of phytoplankton to iron limitation. This information will be particularly useful in the future due to projected alterations in desert dust supply^49,91^ and thus, to iron availability in the ocean, with potential impacts on carbon sequestration and climate. Finally, application of the heme *b* method in oceanic regions other than the Atlantic Ocean would enhance the estimates of the magnitude of the biogenic iron pool and expand our knowledge on phytoplankton physiological status in relation to iron.

## Methods

### Sampling

During all research cruises sampling of seawater was performed by a stainless steel CTD rosette equipped with Niskin bottles for the determination of heme *b*, POC and chl *a*. We sampled 279 stations and 4 to 6 different depths per station (typically down to 300 m deep) in order to study heme *b* abundance in the Atlantic Ocean.

### Heme *b*

Marine particulate material (>0.7 μm) was collected after filtration of seawater on glass fiber filters (GF/F, pore size 0.7 μm, MF300, Fisherbrand) for the determination of heme *b*. The filters were stored at −80 °C prior to analysis. Heme *b* was quantified by High Performance Liquid Chromatography (HPLC) – Diode Array Detection (DAD) – Electrospray Ionisation (ESI) Mass spectrometry (MS) after extraction by 2.5% w-v Octyl β-D-glucopyranoside-OGP solution (Sigma-Aldrich, ≥98% GC Grade)^80,92^. Regarding the analytical procedure and the instruments used, we followed the protocol described by Gledhill (2007) for the samples from the CD173 cruise and Gledhill (2014) for the samples from cruises D346, D361 and M124. Good agreement was obtained between the two analytical methods^92^. Slight modifications were applied to the analysis of the samples from GEOVIDE and M121 as described in Louropoulou *et al*.^36^.

Quantification of heme *b* for D346, D361, GEOVIDE, M121 and M124 was performed from the MS data on a m/z ratio of 616.122, since the PDA detector is known to have interferences from other pigments co-eluting with heme *b* and absorbing at a wavelength of 401 nm^92^. The analytical detection limits were defined as three times the standard deviation of the lowest calibration standard for each of the methods followed and were 1.57 nM (CD173), 190 pmol heme *b* L^−1^ (D346, D361 and M124) and 32 pmol heme *b* L^−1^ (GEOVIDE and M121). Heme *b* could not be detected in the blank extraction solution in either utilised method.

### Particulate organic carbon and chlorophyll *a*

Seawater was filtered through pre-combusted GF/F for the determination of POC and acidified either with sulphurous acid - H_2_SO_3_ (cruises D346, D361, M121, M124) or with hydrochloric acid - HCl (GEOVIDE) for the removal of inorganic carbon following published protocols^93,94^. POC in samples and several blank filters was quantified by elemental NC analyser using acetanilide as the calibration standard^93^.

Chlorophyll *a* was determined for the cruises CD173, D346, D361 and M124 via fluorometry after extraction with 90% (v:v) acetone in the dark^95^. For the GEOVIDE and M121 cruises, chlorophyll *a* was determined after extraction with 100% methanol and sonication followed by High Performance Liquid Chromatography quantification^96^.

### Data handling

Data processing, statistical analysis and visualization were carried out using R Statistical Software^97^ (R Core Team 2016, https://www.R-project.org). The SML was calculated from the CTD data (obtained by sensors on the rosettes) using the temperature-based criterion (ΔT = 0.5 °C)^98^. We checked the normality of the data distribution by applying the Shapiro-Wilk test which showed a non-normal distribution for all parameters (p < 0.05). Hence, we used the Kruskal–Wallis test for analysis of variance, the Wilcoxon rank sum test to identify differences in the distributions in and below the SML and the Spearman’s rank correlation to check the association between parameters. Surface and section plots were produced using the Ocean Data View^99^ v.4.7.9 (Schlitzer, R. Ocean Data View. 2018).

## Supplementary information

Supplementary information.

## Data Availability

Heme *b* and supporting data are available on the British Oceanographic Data Centre, http://www.bodc.ac.uk/.
