# Supplementary material for: Heme b distributions through the Atlantic Ocean: evidence for “anemic” phytoplankton populations
Source: Sci Rep. 2020 Mar 12;10:4551. doi: 10.1038/s41598-020-61425-0 (PMC7067765; doi:10.1038/s41598-020-61425-0)
Supplement: Supplementary file 1 — Supplementary information. [file 41598_2020_61425_MOESM1_ESM.pdf]

Supplementary Information

Heme *b* distributions through the Atlantic Ocean: evidence for "anemic" phytoplankton populations

Evangelia Louropoulou[1,2*], Martha Gledhill[1], Eric P. Achterberg[1], Thomas J. Browning[1], David J. Honey[3], Ruth A. Schmitz[2], Alessandro Tagliabue[4]

[1] GEOMAR Helmholtz Centre for Ocean Research Kiel, Kiel, Germany
[2] Institute for General Microbiology, Christian-Albrechts-Universität, Kiel, Germany
[3] School of Ocean and Earth Science, University of Southampton, Southampton, UK
[4] School of Environmental Sciences, University of Liverpool, Liverpool, UK
*elouropoulou@geomar.de

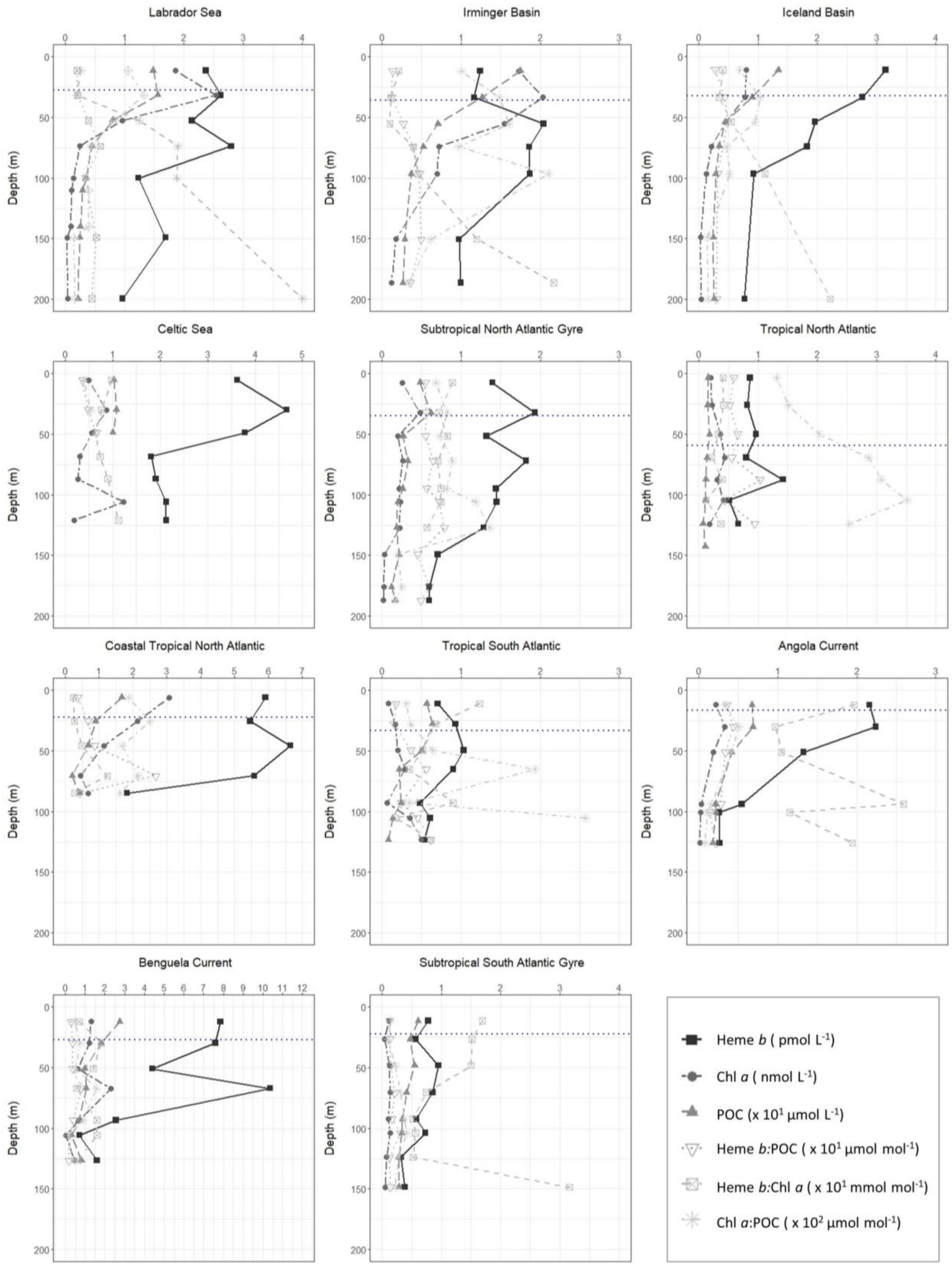

Figure S1. Average depth profiles for heme *b,* chlorophyll *a* (chl *a*), particulate organic carbon (POC), heme *b*: POC, heme *b*:chl *a* and chl *a*:POC. Shapes represent the average value of each parameter in each depth horizon from 0 down to 200m. The dotted line indicates the mixed layer depth (MLD). Each panel represents one oceanographic region; Labrador Sea (LS), Irminger Basin (IrmB), Iceland Basin (IceB), Celtic Sea (CS), Subtropical North Atlantic Gyre (SNAG), Tropical North Atlantic (TNA), Coastal Tropical North Atlantic (CTNA), Tropical South Atlantic (TSA), Angola Current (AnC), Benguella Current (BnC) and Subtropical South Atlantic Gyre (SSAG). The figure was produced using R Statistical Software v.1.0.136 (R core team, 2016, https://www.R-project.org).

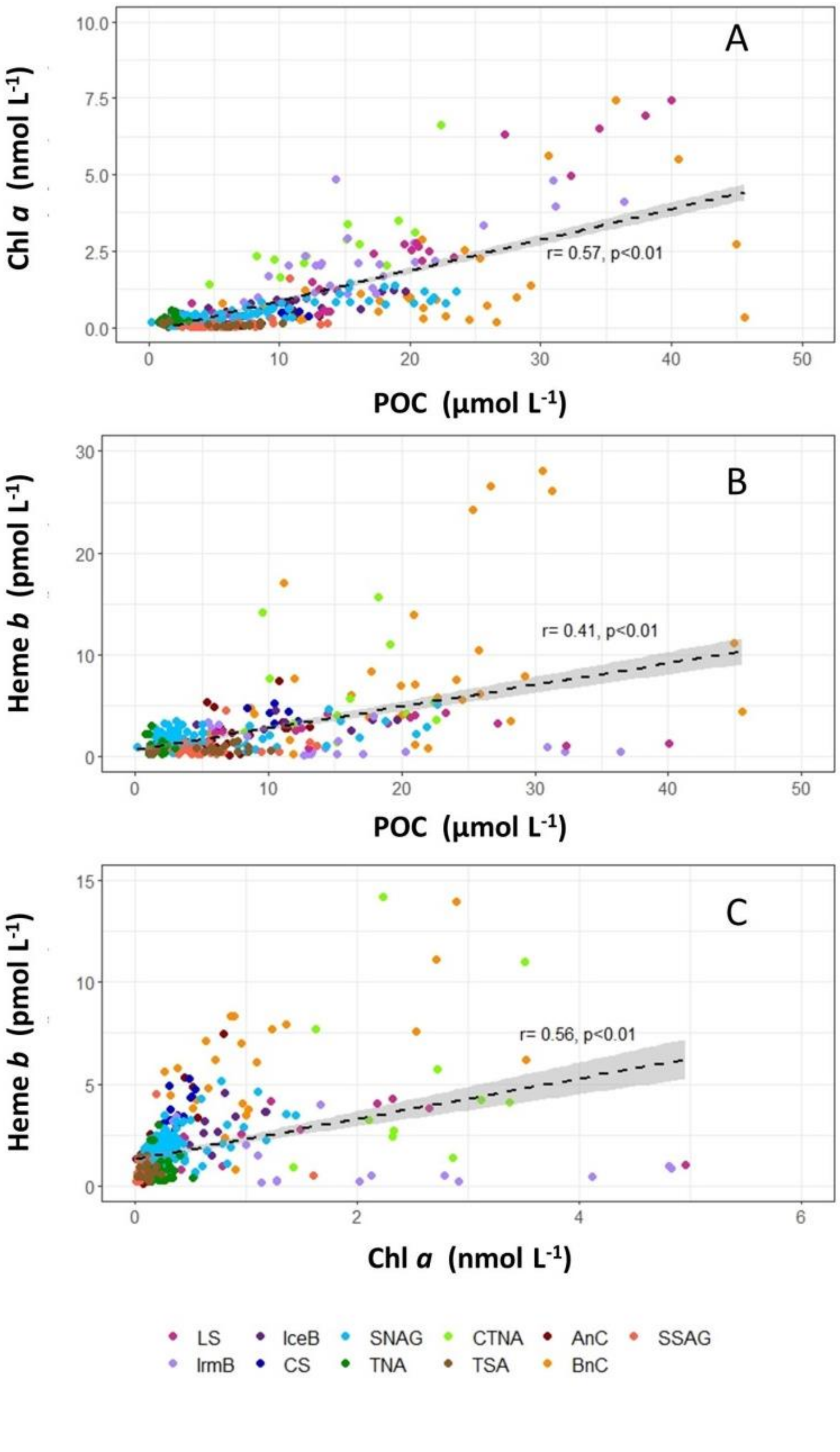

Figure S2. Scatter plots of A) chlorophyll *a* (chl *a*) versus particulate organic carbon (POC), B) heme *b* versus POC, and C) heme *b* versus chl *a* for the surface mixed layer (SML). Dots indicate data points and colours indicate the oceanographic regions; Labrador Sea (LS), Labrador Sea (LS), Irminger Basin (IrmB), Iceland Basin (IceB), Celtic Sea (CS), Subtropical North Atlantic Gyre (SNAG), Tropical North Atlantic (TNA), Coastal Tropical North Atlantic (CTNA), Tropical South Atlantic (TSA), Angola Current (AnC), Benguella Current (BnC) and Subtropical South Atlantic Gyre (SSAG). Letter annotations indicate the correlation coefficients after Spearman's rho rank test. Irminger Basin (IrmB) and the subtropical South Atlantic (SSAG) were excluded from the correlations. The figure was produced using R Statistical Software v.1.0.136 (R core team, 2016, https://www.R-project.org).

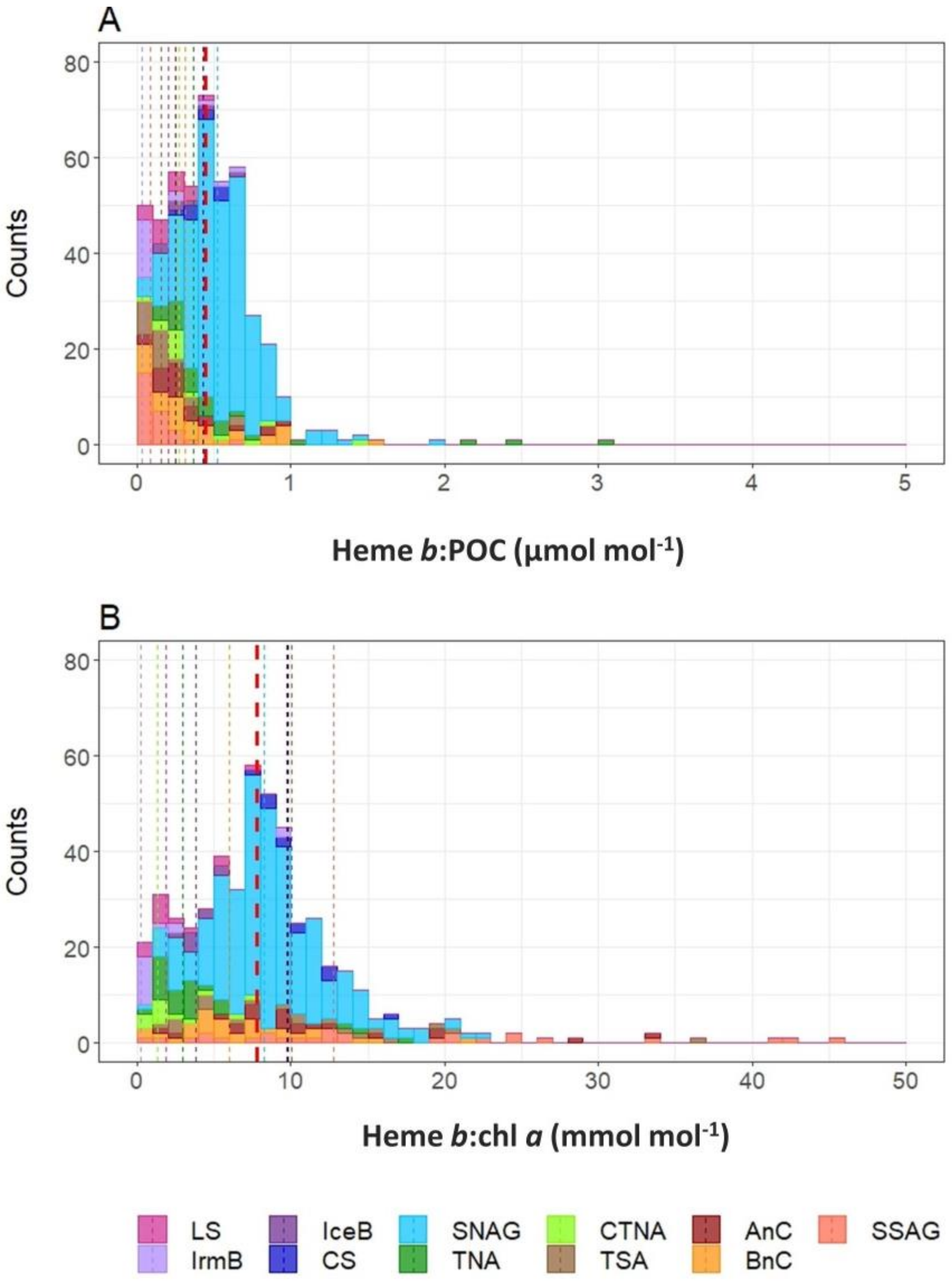

Figure S3. Histograms of A) heme *b*:POC (n=468), and B) heme *b*: chl *a* (n=491) distributions in the surface mixed layer (SML) of the various regions of the Atlantic Ocean obtained from the research cruises from 2005 to 2015. Colours indicate the oceanographic regions; Labrador Sea (LS), Labrador Sea (LS), Irminger Basin (IrmB), Iceland Basin (IceB), Celtic Sea (CS), Subtropical North Atlantic Gyre (SNAG), Tropical North Atlantic (TNA), Coastal Tropical North Atlantic (CTNA), Tropical South Atlantic (TSA), Angola Current (AnC), Benguela Current (BnC) and Subtropical South Atlantic Gyre (SSAG). The thick red dashed line marks the median of the each distribution while the coloured thinner dashed lines indicate the medians of each oceanographic region. The figure was produced using R Statistical Software v.1.0.136 (R core team, 2016, https://www.R-project.org).
